# Wet tropical climate in SE Tibet during the Late Eocene

**DOI:** 10.1038/s41598-017-07766-9

**Published:** 2017-08-10

**Authors:** Philippe Sorrel, Ines Eymard, Philippe-Herve Leloup, Gweltaz Maheo, Nicolas Olivier, Mary Sterb, Loraine Gourbet, Guocan Wang, Wu Jing, Haijian Lu, Haibing Li, Xu Yadong, Kexin Zhang, Kai Cao, Marie-Luce Chevalier, Anne Replumaz

**Affiliations:** 1 Université Lyon 1, ENS de Lyon, CNRS, UMR 5276 LGL-TPE, F-69622 Villeurbanne, France; 2 Department of Earth Sciences, University of Geneva, Geneva, Switzerland; 3 Laboratoire Magmas et Volcans, Université Blaise Pascal – CNRS – IRD, OPGC, Clermont-Ferrand, France; 4 School of Earth Sciences, China University of Geosciences, Wuhan, 430074 China; 5 Center for Global Tectonics, China University of Geosciences, Wuhan, 430074 China; 6 Institute of Geology, Chinese Academy of Geological Sciences, Beijing, China; 7 ISTerre, Université Grenoble Alpes, CNRS, Grenoble, France

## Abstract

Cenozoic climate cooling at the advent of the Eocene-Oligocene transition (EOT), ~33.7 Ma ago, was stamped in the ocean by a series of climatic events albeit the impact of this global climatic transition on terrestrial environments is still fragmentary. Yet archival constraints on Late Eocene atmospheric circulation are scarce in (tropical) monsoonal Asia, and the paucity of terrestrial records hampers a meaningful comparison of the long-term climatic trends between oceanic and continental realms. Here we report new sedimentological data from the Jianchuan basin (SE Tibet) arguing for wetter climatic conditions in monsoonal Asia at ~35.5 Ma almost coevally to the aridification recognized northwards in the Xining basin. We show that the occurrence of flash-flood events in semi-arid to sub-humid palustrine-sublacustrine settings preceded the development of coal-bearing deposits in swampy-like environments, thus paving the way to a more humid climate in SE Tibet ahead from the EOT. We suggest that this moisture redistribution possibly reflects more northern and intensified ITCZ-induced tropical rainfall in monsoonal Asia around 35.5 Ma, in accordance with recent sea-surface temperature reconstructions from equatorial oceanic records. Our findings thus highlight an important period of climatic upheaval in terrestrial Asian environments ~2–4 millions years prior to the EOT.

## Introduction

Earth’s climate gradually cooled from greenhouse to icehouse climatic conditions in the Late Eocene, leading to the Eocene–Oligocene Transition (EOT) at ~33.7 Ma and permanent polar glaciation in Antarctica^1–3^. This climatic deterioration was punctuated by abrupt and short-lived cooling and warming events in the time period referred as the Late Eocene « Doubthouse » (LED)^4^, which has long been well-documented in oceanic records (e.g., refs 1, 5). Very recently, a new sea-surface temperature (SST) record revealed that an unequivocal tropical warming at equatorial oceanic latitudes initiated 4 million years prior to Antarctic glaciation, leading to an increase of heat accumulation in the low latitudes and a prominent meridional ocean reorganization predating the EOT^6^. Nevertheless, the extent to which the Late Eocene warming impacted low-latitude climates, in particular in remote continental areas, remains largely unknown. Given its crucial role in warm climates of the Quaternary, one of paleoclimatology’s grandest challenge is to decipher whether the dynamics of the Intertropical Convergence Zone (ITCZ), the dominant feature of atmospheric dynamics in the tropics, could be involved in episodes of prominent climatic change in deep time. In particular, what were the main forcings of climatic variability during this warming interval preceding the EOT? In terrestrial sedimentary records, the LED is recognized within the Xining basin (NE Tibet) by a progressive, stepwise aridification^7, 8^ linked to the retreat of the Paratethys epicontinental sea and a persistent monsoon-like pattern in tropical Asia since at least 40 Ma^9^. However, the paucity of paleoenvironmental data in the tropical latitude zone, especially from well-dated terrestrial records, hampers the evaluation of the response of continental settings to global climatic change during the LED, much needed for the understanding (and modelling) of processes leading to the transition from greenhouse to icehouse conditions. To address this issue, we present the first well-dated sedimentary (terrestrial) record revealing foremost paleoenvironmental change at tropical latitudes (19–21°N) in SE Tibet during the LED. In light of a revised regional Cenozoic stratigraphy and a comprehensive analysis of sedimentary facies, we show that fluvial-palustrine-lacustrine successions of the Jianchuan basin unveil a progressive increase in tropical humidity in SE Tibet, sheding light on atmospheric circulation patterns in SE Asia during the Late Eocene.

## Geological setting and stratigraphy

**Figure 1:**
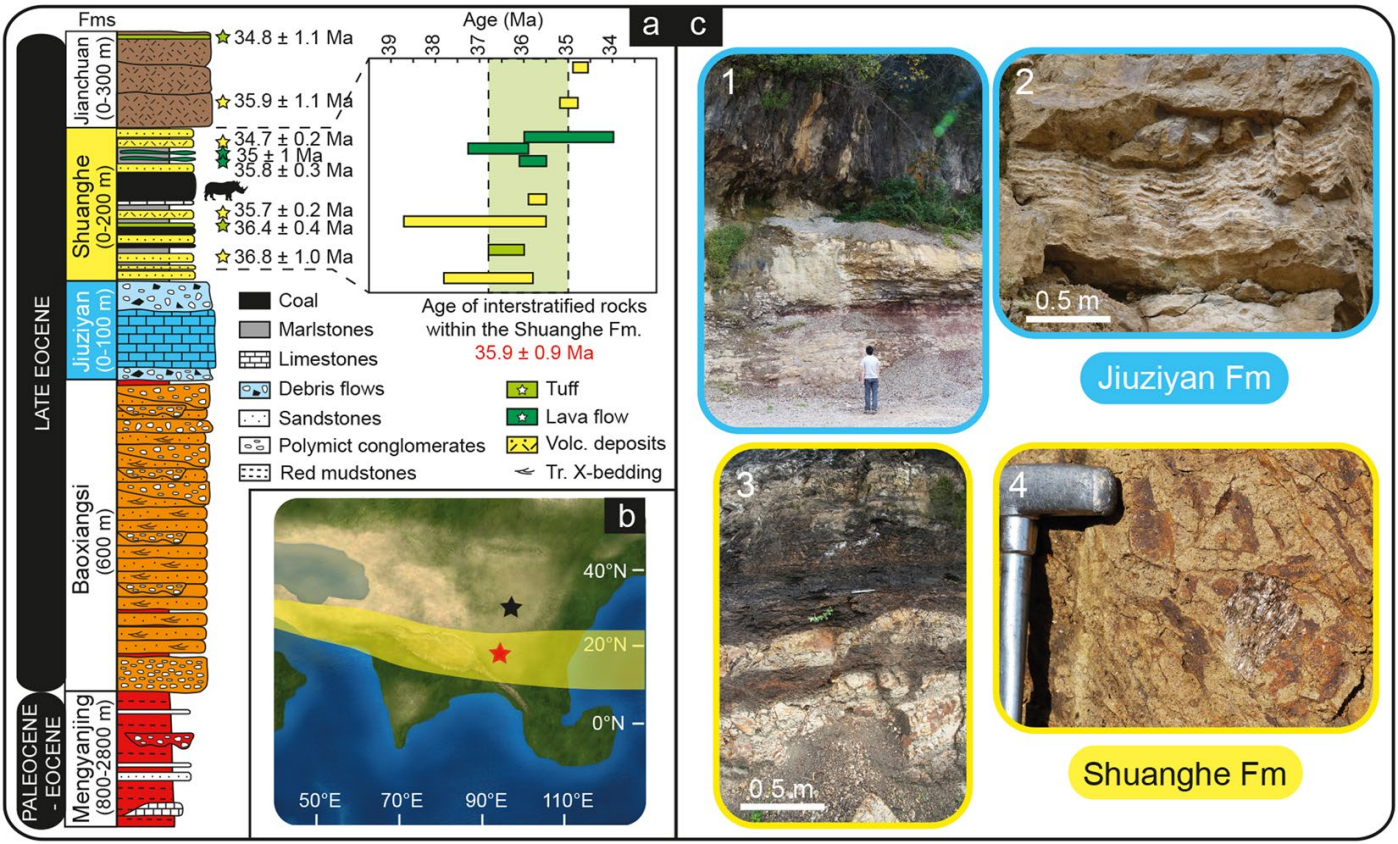
The Late Eocene Jianchuan basin in SE Asia: paleogeography, stratigraphy and sedimentology. (**a**) Revised Cenozoic stratigraphy for the Jianchuan basin based on new field data including new stratigraphic and structural interpretations^11^. New ages from volcanic and volcanoclastic deposits (interbedded within sandstones and coal deposits) indicate that the Shuanghe Fm. formed at 35.9 ± 0.9 Ma. Blue and yellow shadings for Jiuziyan and Shuanghe Fms., respectively, refer to Fig. 1c. Volc. deposits: volcanic deposits; Tr. X-bedding: trough cross-bedding. Not to scale. (**b**) Late Eocene paleogeography and paleotopography of SE Tibet and the Asian mainland (modified from Licht *et al*.^9^). Paleogeographic reconstruction used in the Eocene–Oligocene transition (34 Ma ago) simulations of Lefebvre *et al*.^51^. Red star refers to our study site in SE Tibet (Jianchuan basin). Black star corresponds to the Xining basin in NE Tibet^9^. The yellow shaded area refers to a tentative reconstruction of the possibly more northern (summer) position of the ITCZ in SE Asia between ~37 and ~35 Ma. (**c**) Sedimentary facies of the Jiuziyan (blue border) and Shuanghe (yellow border) Fms^1^. The Jiuziyan Fm. consists of a thickening-up alluvial-palustrine succession from basal red mudstones (facies Mu) and siltstones (facies Si) to upper fluvio-palustrine-lacustrine deposits^2^; Stromatolitic limestones, which typify the Jiuziyan Fm^3^; The Shuanghe Fm. consists here of sandstones regularly interbedded with coal deposits and thin carbonate layers formed in a swampy-like environment^4^; Close-up on the bioclatic sandstones containing abundant vegetal remains (wood fragments and phytoclasts).

**Figure 2:**
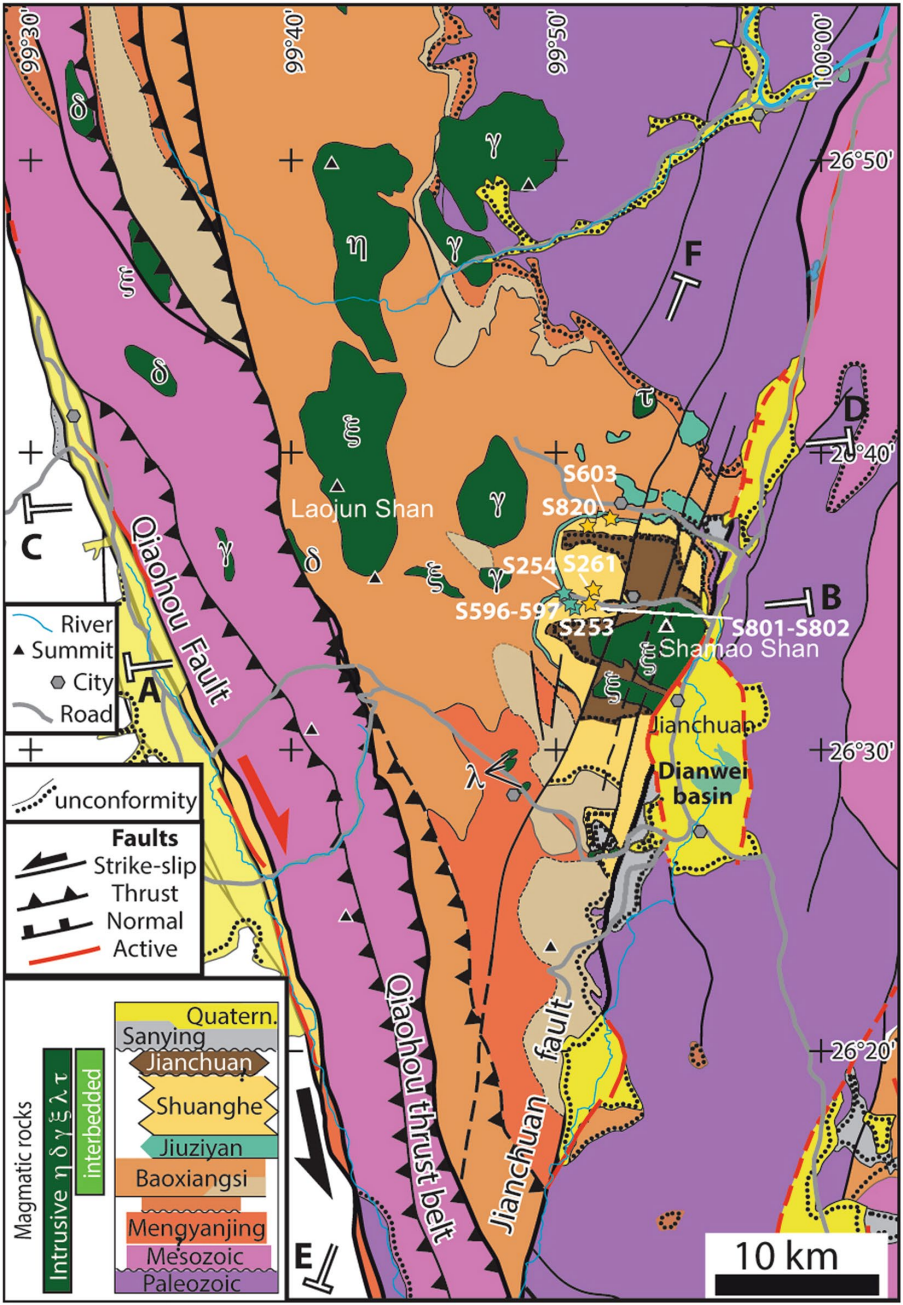
Revised Cenozoic stratigraphy for the Jianchuan basin and structural map of the Jianchuan basin (modified after 11). The inset in the lower-left corner depicts the geometrical and temporal sedimentary relationships between the formations, with undulations corresponding to angular unconformities. Sites S253, S254, S261, S596, S597, S603, S801, S802 and S820 (stars) refer to locations referred to in the text and figures. Magmatic rocks lithology: ξ syenite, δ diorite, γ granite, η norite, τ trachyte. Quatern: Quaternary. The lithology of Paleogene formations from the Jianchuan basin is detailed in the text. Base map obtained from Bureau of Geology and Mineral Resources of Yunnan Province^52^. This figure was generated using MAPublisher 9.9 (http://www.avenza.com/mapublisher) under Adobe Illustrator CS6 (www.adobe.com/).

**Figure 3:**
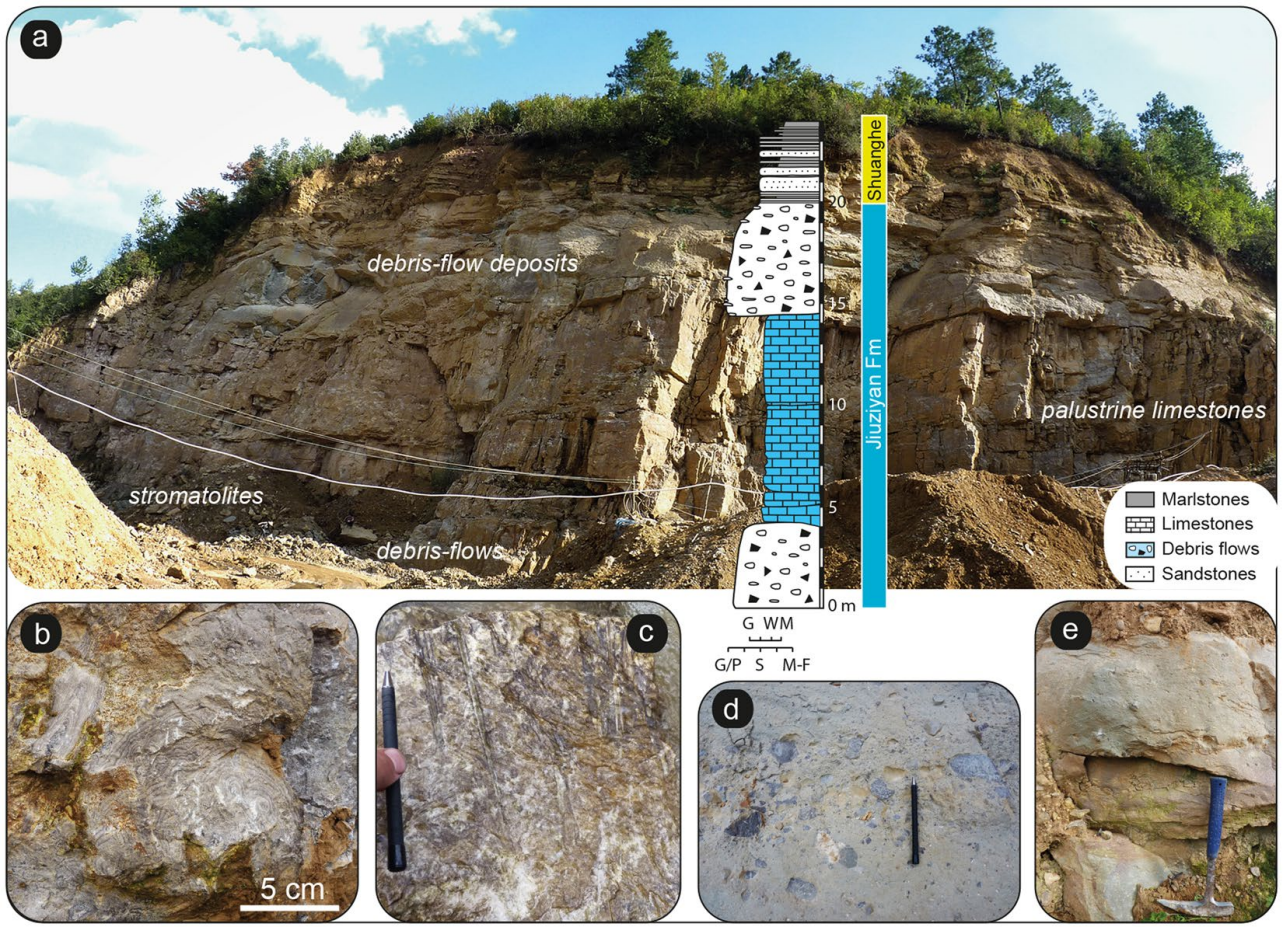
The fluvio-palustrine-lacustrine succession of the Jiuziyan Fm. (site S254). (**a**) Sedimentary succession showing the alternation between palustrine limestones and overlying massive debris-flow deposits (e.g., matrix-supported conglomerates) associated to massive high-energy floods in palustrine-sublacustrine settings. The abbreviations used for the textural scale are: ‘M-F’: marls and fines (siliciclastic mudstones); ‘S’: sands; ‘G-P’: gravels and pebbles; ‘M’: mudstones; ‘W’: wackestones; ‘G’: grainstones. (**b**) *In situ* decimeter-sized domical stromatolites (facies Str). Stromatolites grew in calm, ponded areas of the palustrine-lacustrine system. (**c**) Boundstone of stems (e.g., phytoherm tufa of stems, facies Phs). (**d**) Debris-flow deposits (calcisiltites/calcilutites) including angular to subangular carbonate lithoclasts, as floating rafts in argillaceous calcisiltites. (**e**) Massive calcisiltites/calcilutites (with rare carbonate lithoclasts) corresponding to high-energy floods in palustrine-sublacustrine areas.

The well-preserved sedimentary succession of the Jianchuan basin (NW Yunnan, China) affords a unique perspective to study the sedimentary signature of environmental and climatic processes on the SE margins of the Tibetan Plateau (Fig. 1). The paleolatitudes of the Jianchuan basin for the Late Eocene (19–21°N) were calculated based on APWP (paleomagnetic apparent polar wander path) of East Asia^10^. Late Eocene sedimentation took place in an intermontane basin including alluvial fan, palustrine and sublacustrine environments, fed by alluvial fans draining Paleozoic and Mesozoic bedrocks to the east (Fig. 2). In the Jianchuan basin, Paleogene basin-fill strata are composed of the Mengyanjing Formation (Fm.), the Baoxiangsi Fm., the Jiuziyan Fm., the Shuanghe Fm. and the Jianchuan Fm. (Figs 1 and 2, see also Supplementary Figure S1). The Mengyanjing Fm. (800–2800 m) consists of red mudstones and siltstones, with local occurrence of sandstones. The Baoxiangsi Fm., commonly **~**600 m thick, is made of a thick series of sandstones, siltstones and conglomerates. In many places, beds are nearly horizontal and conformably overlie the Mengyanjing Fm. Given the presence of conglomerate levels, the depositional environment was proximal to a local palaeo-relief, in particular to the northeast and east basin-margin from which sources likely originated (Fig. 2)^11^. The Jiuziyan Fm. and Shuanghe Fm. are usually described as resting unconformably on all underlying units, however we did not find any clear angular unconformity. The Jiuziyan Fm. (0–100 m thick) consists of a multi-storey carbonate succession interbedded with massive matrix-supported conglomerates and argillaceous calcisiltites (Fig. 1). Locally, the base of the Jiuziyan Fm. is erosive on the underlying red mudstones (Mengyanjing Fm.) while at other sites the succession shows a regular transition without any clear angular unconformity. The Jiuziyan Fm. appears mostly restricted to the northeastern part of the Jianchuan basin. The Shuanghe Fm. (0–200 m thick), which overlies conformably the Jiuziyan Fm. (Fig. 3), extends on the eastern side of the basin. It consists of poorly consolidated sandstones and marlstones regularly interbedded with coal deposits (that thicken upwards) and occasional lava flows, tuff and volcano-sedimentary levels. The Shuanghe Fm. is cut by intrusive rocks (mostly lamprophyres), which form numerous sills in the coal levels. The Jianchuan Fm. (0–300 m thick) mostly consists of volcanoclastic deposits, and it has been interpreted as Neogene volcanoclastics. We further refer to ref. 11 for a more comprehensive description of the depositional environments related to these five main formations, as well as for a thorough reapparaisal of the Cenozoic Jianchuan basin stratigraphy. The newly revisited stratigraphy in the Jianchuan basin allows a re-evaluation of time constraints on basin infill during the Late Eocene^11^. Indeed, new ages obtained on zircons (U/Pb) and biotite (Ar/Ar) from volcanites interbedded within sandstones and coal deposits imply that the Shuanghe Fm. deposited between ~37 and 34.7 Ma, with a mean weighted average age of 35.9 ± 0.9 Ma^11^ (see also Fig. 1). In addition, well-preserved mammal dental remains recently discovered in the Shuanghe Fm. (site S603, Fig. 2) belong to a giant hippo-like amynodontid rhinoceratoid far restricted to the Late Eocene Ergilian interval (37.2 to 33.9 Ma) in Asia^11^. The combination of geochronological and paleontological age constraints^11^ therefore strictly imparts a Late Eocene age to the Jiuziyan, Shuanghe and Jianchuan Fms. (Fig. 1). Here we focus on the sections in the eastern part of the Jianchuan basin, with a particular emphasis on the Jiuziyan and Shuanghe Fms. deposited between ~37 and 35 Ma.

## Results and Interpretations

The statigraphic record dated between ~37 and 35 Ma (e.g., Jiuziyan and Shuanghe Fms) can be split into two parts based on its lithological and facies characteristics. The lower part, corresponding to the Jiuziyan Fm, contains marlstones, sandstones, siltstones, matrix-supported conglomerates and a large dominion of limestone deposits (Fig. 1c; see also Supplementary Figures S2 and S3). The upper part, corresponding to the Shuanghe Fm., contains marlstones intertwined with sandstones and sees the late-stage occurrence of coal deposits (Fig. 1c). Fifteen lithofacies are identified within the Jiuziyan and Shuanghe Fms. The macroscopic and microscopic features of these facies (geometry, texture, sedimentary structures and biogenic components), along with their interpretation as depositional environments, are summarized in Supplementary Table 1 (see Supplementary Information). References to works that involve similar facies and depositional environments are included in Supplementary Table 1.

**Figure 4:**
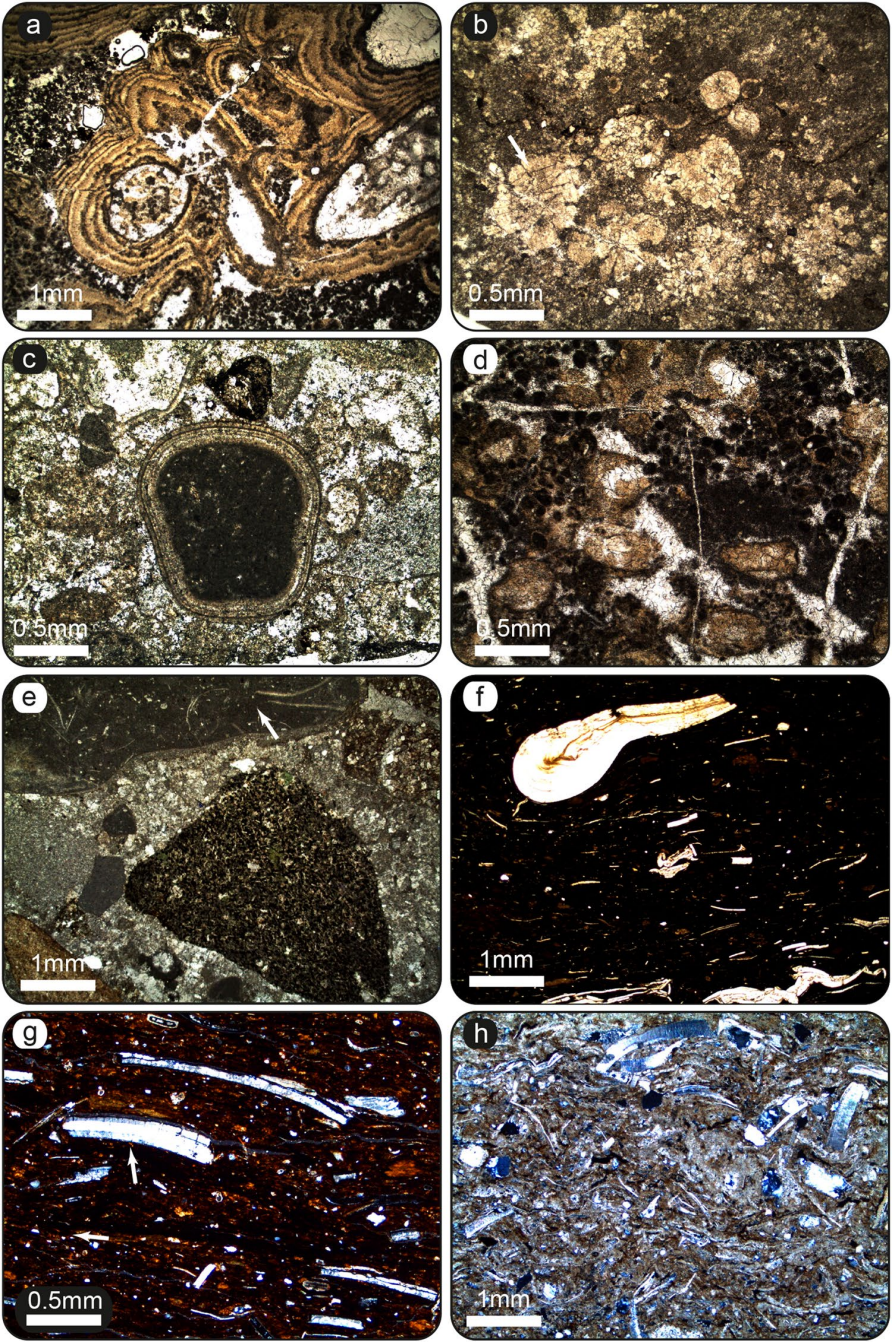
Palustrine-lacustrine microfacies of the Jiuziyan Fm. (**a**–**d**) and Shuanghe Fm. (**e**–**h**). See Fig. 2 for location of sites. (**a**) Microbial deposits encrusting calcified macrophyte stems (facies Phs). The coating consists of an alternation of light and dark laminae as in stromatolites. Note the micropeloidal matrix filled with microspar cement (Sample J194, site S596). This facies typifies primary palustrine-lacustrine deposits before undergoing pedogenesis. (**b**) Pedogenic features (caliche, facies Cal). Calcified root structures in cross section made of individual calcified root cells, creating typical *Microcodium* structure (arrow) (Sample J225, site S254). (**c**) Granular limestone (facies Lgr) composed of both intraclasts and extraclasts within interstitial microspar cement. Note the large oncoid consisting of a Paleozoic/Mesozoic nucleus (carbonate extraclast) and a sub-mm cortice made of alternating calcite spar to microspar laminae (Sample J158g, site S254). (**d**) Nodular micropeloidal and intraclastic limestones. Note the presence of caliches such as circumgranular dessication cracks, filled with microspar cement (Sample J194b, site S596). (**e**) Granular limestone including extraclasts (marine Paleozoic/Mesozoic packestone – arrow, and igneous rock fragments) filled by microspar cement (Sample J171, site S254). (**f**) Palustrine/lacustrine facies (facies Co). Coal layer containing abundant vegetal remains (Sample J157, site S253). (**g**) Coal layer (facies Co) containing abundant vegetal remains (arrow) (Sample J169, site S261). (**h**, cross-nicols) Primary lacustrine facies (facies Lorg). Wackestone with amalgamated shell fragments within a micritic matrix (Sample J285A, site S820).

The most represented facies in the Jiuziyan Fm. are those of massive matrix-supported conglomerates (facies Cong, Fig. 3a,d) and phytoclastic floatstones/wackestones (facies Lphy; Supplementary Figures S2, S4), which commonly occur associated with minor boundstones of stems (e.g., phytoherm tufa of stems; facies Phs; Figs 3c and 4a). Intraclastic and micropeloidal, oncoidal and granular limestones are also common in this facies (facies Lim, Lgr; Fig. 4c–e and Supplementary Figure S4). Stromatolites are restricted to this unit and constitute tabular and lenticular strata (facies Str; Fig. 3b and Supplementary Figures S3, S4), at places forming domes, centimetres to 1 m thick (Fig. 3b). Stromatolitic layering shows flat-laminated, undulatory and less common mammelar, columnar-layered and pseudocolumnar structures. Marlstones (facies Mar) alternating with sandstones showing trough cross-stratification and foreset beds (facies Sa; Supplementary Figures S2, S3) are also present. The most common alluvial facies consists of red mudstones (facies Mu; Fig. 1c), which evolves upwards to siltstones (facies Si) interwined with planar-laminated sheet-like sandstones and blue-grey sublacustrine marls (Fig. 1c). The composition of lithoclasts covers a wide spectrum of carbonate lithologies including limestones and mudstones. Massive matrix-supported conglomerates are mostly composed of polymictic extraclasts of limestones (Fig. 3d). Marine fossil fragments are commonly found within gravel- and sand-sized lithoclasts of limestones (Fig. 4e and Supplementary Figure S4). The most abundant facies in the Shuanghe Fm. are those of sandstones (facies Sa) and calcisiltites (Csl) intercalated within grey marlstones (Mar). Sandstones (Sa) commonly show trough cross-stratification (Supplementary Figure S2). Bioclastic sandstones (facies Sb; Fig. 1c) occur intermingled with organic-rich bioclastic limestones (facies Lorg, Fig. 4h) and definite coal layers (facies Co; Figs 1c and 4f,g and Supplementary Figures S2 and S5). Coal deposits are locally exploited in mines (S261; Fig. 2). Bioclastic limestones mostly consist of floatstones of vegetal remains (Supplementary Figure S2).

### Facies and facies associations

The facies detailed in Supplementary Table 1 shape vertical associations that illustrate the superposition of sediments deposited in laterally related sub-environments. Four main facies associations (FAs) were recognized in this study.

#### Fan-delta edge – shallow lacustrine association (FA1)

This facies association (FA1a) is characterized by grey marlstones deposited in calm shallow waters (facies Mar) intertwined with sandstones showing trough cross-bedding and foreset beds (Supplementary Figure S3). Occasional climbing-ripple cross laminations can be identified (facies Sa), which are typical of superimposed migrating ripples formed when deposition exceeds migration in aqueous hyperconcentrated flows^12^. Locally, these marlstones pass upwards into thick-bedded matrix-supported conglomerates, most often structureless, in which disorganized pebble-sized lithoclasts occur as floating rafts in the argillaceous calcisiltites (facies Cong; Fig. 3a,d). Carbonate lithoclasts are predominantly subrounded in shape (Supplementary Figure S2), although some massive matrix-supported conglomerate layers contain preferably angular to subangular lithoclasts (Fig. 3d). Pebbly carbonate calcisiltites are interpreted as having been deposited by concentrated (or high-density) underflows, which were developed from the cohesive debris flows as a result of the progressive entrainment of water and sediment dilution during the course of downward transport^12–15^ to the sublacustrine system. Similar deposits have been described in Eocene lacustrine systems of north China^16^. All of these features suggest that this facies association was deposited in a fan-delta edge interfingered with sublacustrine areas, with frequent debris flows released from updip alluvial fans as a result of catastrophic flooding. Carbonate alluvial fans are fan-delta systems, as inferred from upward-coarsening successions^17^ (Fig. 1c).

FA1b lacks the matrix-supported conglomerates and represents deposition of unconfined flows in sublacustrine areas. Argillaceous calcisiltites (facies Csl; Supplementary Figure S3) delivered after a rapid flooding are commonly interbedded within grey marls. Cm-thick to dm-thick sandstones intertwined with marls also occur in FA1b (Supplementary Figure S1). Its depositional significance is the same of that of FA1a but the concentrated (or high- density) underflows likely occurred under less energetic conditions.

#### Distal alluvial fan association (FA2)

This facies association consists of red mudstones typical of floodplain deposits (facies Mu; Fig. 1c), intercalated with decimeter-thick sheet-like siltstones (to sandstones) interpreted as overbank (or crevasse-splay) deposits on an alluvial plain, as described elsewhere^18, 19^. The alluvial plain accreted from flood events, with water overflowing nearby channels. Rare carbonate-fill channels are also observed. This process progressively generated a mostly flat-convex, thickening-up succession (e.g., overbank lobes) made of lenticular siltstone bodies (facies Si) evolving to thick-bedded calcisiltites (facies Csl) deposited in sublacustrine areas. It occurs in the distal part of the alluvial fans, and often interfingers with grey marlstones (Fig. 1c). The presence of caliches (Facies Cal), especially in basal red alluvial mudstones implies that these deposits temporarily were formed subaerially^20^. The alveolar septal structures, circumgranular cracks in caliches show evidence of apparent microbial activity around roots during pedogenic processes^20, 21^, commonly occuring under intermediate climatic conditions^22^.

#### Palustrine association (FA3)

This facies association documents a typical palustrine environment, in which a dense rooted hydrophilous vegetation developed on the lake margins and adjacent ponded areas. The submerged parts of the hydrophilous vegetation became coated with calcite, locally producing phytoherms of stems (facies Phs; Fig. 3c and Supplementary Figure S2). During high-energy flooding, such as those reported in FA1, the plants were fragmented and deposited *in situ* or in the close vicinity (facies Lphy; Supplementary Figure S2). Rare fluvial channel-fill deposits are observed (Supplementary Figure S3). The phytoclasts (both calcite-coated and uncoated) thus generated would serve as nuclei for the formation of oncoids that would accumulate in shallow channels and littoral lake areas. The development of stromatolites (facies Str) over the intraclastic and oncoidal limestones (facies Lphy and Lim) infers a reduction in the energy conditions. In turn, shallow pools and ponds formed from abandoned channels and the littoral lake areas were densely settled by rooted plants. Similar associations have been reported within fluvial-palustrine environments^23, 24^. The progressive shallowing and dessiccation of the palustrine system generated a great variety of pedogenic features (e.g., caliches; facies Cal; Supplementary Figure S3), which suggest long periods of subaerial exposure as described elsewhere^25^. Circumgranular cracks formed during dessiccation (Fig. 4d); breccias and root traces are common in FA3. Locally, dolomitization of spar cement can be observed, usually associated to circumgranular cracks. Alveolar septal structures and *Microcodium* are also common features in FA3 (Fig. 4b and Supplementary Figures S4, S5). Alveolar septal structures are generally interpreted as calcification related to fungal activity mostly in the vicinity of roots^26^. The presence of alveolar septal structures indicates that root and microbes contributed to the fragmentation of the original (palustrine) deposits^27, 28^. Frequent occurrences of calcified cells typical of *Microcodium* is another evidence for pedogenesis in palustrine environments.

#### Swamp-like alluvial-palustrine association (FA4)

FA4 is characterized by abundant coal-bearing and intertwined thin limestone deposits (facies Lorg; Fig. 4f–h and Supplementary Figures S2, S3 and S5) regularly interbedded within sandstones (facies Sb; Supplementary Figures S2, S3). Limestone deposits mostly consist of floatstones of vegetal remains (Supplementary Figure S2) and wackestones/packestones of bivalve (Fig. 4h) and gastropod fragments enriched in organic matter. Coal deposits are exploited in local mines (e.g., S261; Fig. 2 and Supplementary Figure S2). The presence of aquatic organisms within carbonate layers, as well as the formation of coal deposits occurring towards the top of the Shuanghe Fm. suggests the development of a swampy-like environment. This facies association represents sedimentation in shallow sublacustrine and littoral pool environments^22, 29^ under a semi-humid to humid climatic conditions.

### Intermediate climate conditions during the deposition of the Jiuziyan Fm

Superimposed on basal red alluvial beds (Mengyanjing Fm.), the deposition of the Jiuziyan Fm. (site S254 in Fig. 2) took place in a shallow carbonate palustrine-lacustrine (e.g., near-shore) system with associated palustrine fringes (FA3). The system received abundant alluvial (e.g., fan-deltaic) sediment input from distal carbonate alluvial fans, i.e., massive matrix-supported conglomerates generated by unconfined flows in sublacustrine areas (Fig. 3). A wide array of carbonate facies including floatstones, wackestones-packestones containing fragments of macrophytes, intraclasts, oncoids, peloids, ostracods and caliches evidencing subaerial exposure typifies palustrine-lacustrine deposits^18^ (Fig. 4, Supplementary Table 1 and Supplementary Figures S2–S5). Ponds and pooled areas bounded by abundant hydrophilous vegetation were likely loci of formation of wackstones/packestones to floatstones of phytoclasts, ostracods and various intraclasts/extraclasts (Fig. 4d,e). Shallow palustrine areas with hygrophytic plants were in turn sites for boundstone formation (e.g., phytoherm tufas of stems; Fig. 3c), from where phytoclasts could be reworked during flood events. Therefore, facies associations of the Jiuziyan Fm. exhibit congruences with those recognized in other palustrine-lacustrine deposits^18, 21, 30, 31^. The calcium-bicarbonate composition likely favored the precipitation of calcite on the submerged parts of plants (Fig. 4a). This implies an abundant supply of bicarbonate-rich surface water inputs from a carbonate source area, likely originating from basin-margin Paleozoic and Mesozoic bedrocks (and outflow of related aquifers) located to the east of the basin (Fig. 2), as also inferred from occurrences of marine carbonate lithoclasts during flood events (Fig. 4e and Supplementary Figure S3). These inputs would have supplied calcium-rich water to the loci of microbial and associated carbonate palustrine-lacustrine deposits as also exemplified in present-day Walter Lake, USA^32^. The deposition of massive matrix-supported conglomerates (e.g., debris flows) of eastern provenances therefore implies the existence of adjacent elevated settings from the uplifted areas of the basin, less than 10 km to the east^11^. Weathered carbonate substratum from Paleozoic and Mesozoic bedrocks could have thus provided both the clastic and dissolved carbonate loads to the palustrine-lacustrine environment in the Jianchuan basin. From a climatic viewpoint, carbonate alluvial fan-deltas and palustrine-lacustrine deposits most likely formed under intermediate climate conditions on the lower slope of the eastern bounding palaeo-relief. Although microbial deposits and caliches can form in various climate conditions, their development is favored in dry subhumid to subarid climate^33^. Evidence for well-developed microbial deposits coupled with the bountiful variability of caliches in palustrine carbonates thus bolsters the view of a semi-arid to sub-humid (e.g., intermediate) climate during the deposition of the Jiuziyan Fm. In turn, debris flows conveying carbonate extraclasts deposited in distal alluvial fan-deltas were associated to increased rainfalls (mostly flash flooding) that intensified erosion of the catchment. Enhanced rainfall activity might be associated to increased tropical storm activity in an Eocene greenhouse climate^34^. The episodicity of flash-flood occurrences in palustrine-sublacustrine areas thus infers fluctuations in surface water discharges (associated to high sediment loads) to the basin, most probably tied to pronounced regional episodic climatic perturbations.

### Wetter climatic conditions from ~35.5 Ma onwards

In the Shuanghe Fm, the palustrine carbonates (Jiuziyan Fm.) were replaced by a sublacustrine (alluvial-lake) system consisting mainly of fine-grained sandstones intertwined within lacustrine marls (FA1b). The extensive presence of plant fossils in fine-grained limestones (Fig. 3c, and Supplementary Figures S2, S5) implies that the climate evolved to a semi-humid to humid climate^35^. Most interestingly, the topmost part of the Shuanghe Fm. consists of coal deposits (Figs 1c and 4f,g and Supplementary Figure S5), with occurences of limestones containing gastropod, ostracod and bivalve shells (Fig. 4h and Supplementary Figure S5) interbedded within sandstones (FA4). The formation of coal layers implies (i) the development of an extensive vegetation in a swampy-like catchment and (ii) abundant precipitation and increased water supplies in the Jianchuan basin, with precipitation always dominating over evaporation^29^. In addition, as the climate humidified, increased precipitation would increase the volume of water and amount of dissolved sediments due to enhanced chemical weathering. That would in turn (due to baffle and trapping effect of a denser vegetation in the catchment) lead to reduced sediment loads, including the coarse lithoclasts, to the basin, as reported elsewhere^35^. Of uttermost relevance is the presence of abundant woody/ligno-cellulosic fragments in the Shuanghe Fm. (Fig. 1c and Supplementary Figure S5) indicating incipient moister conditions around ~35.5 Ma in SE Tibet. The formation of coal in the latter stages of the Eocene therefore heralds the development of swamps at the expense of a semi-arid to semi-humid palustrine system, coevally with the occurrence of pervasive magmatic events around ~35.5 Ma^11^.

## Discussion

Our results reveal a prominent environmental shift from palustrine-lacustrine sedimentary signatures (indicative of semi-arid to semi-humid conditions in the Jiuziyan Fm.) to more humid conditions around ~35.5 Ma, as revealed by regular occurrences of coal-bearing deposits (Shuanghe Fm.), thus providing regional lines of evidence that SE Tibet experienced a marked climatic change associated with amplified precipitation during the Late Eocene. This possibly includes a decline of the dry season duration (although more data would be required to investigate this issue), but more likely enhanced annual precipitation and thus a waning of the continentality in SE Tibet around ~35.5 Ma. Our findings corroborate recent climate simulations arguing for a strong seasonality in summer *vs* annual rainfall at the latitudes of our study site at 40 Ma, but a subsequent decline of the seasonality at ~34 Ma^9^. Consistent with this, previous studies inferred that Late Eocene climate was perennially moist in the tropical to sub-tropical zone of SE Asia^36–38^; in contrast, the onset of widespread aridification was inferred in the Xining basin to the north (NE Tibet) at ~36.6 Ma, both expressed in palynofloral assemblages^39, 40^ and lithofacies variations^7^. Our findings do not exclude that, in conjunction with rapid climatic change during the Late Eocene, long-term tectonic processes also led to regional paleoenvironmental changes. However, recent re-evaluation of Late Eocene paleoelevation estimates relying on stable isotope paleoaltimetry unambiguously yield a 1200 ± 1200 m.a.s.l. paleoelevation for the Jianchuan basin^11^. Revised estimates therefore suggest that the Jianchuan area was most likely at low elevation at ~35.5 Ma, or at least at a lower elevation than today^11^. Collectively, it implies that a significant part of the uplift in Eastern Tibet occurred only after Late Eocene time, thus precluding a pervasive impact of relief evolution on regional climate. In addition, we assume the migration of the Asian continent to the North to be negligible during the Late Eocene^41^. The calculation of paleolatitudes for the Jianchuan basin based on new paleomagnetic data^10^ yield paleolatitudes estimates of 18.8°N ± 4.2° and 20.8°N ± 2.8° at 40 and 30 Ma, respectively. Therefore, the migration of the Asian continent between 40 and 30 Ma stands within the error bars, and can not account for the prominent climatic change observed in this study. Hence, our findings underscore that Late Eocene climate dynamics in SE Tibet most likely responded to large-scale climatic change, rather than to local/regional tectonism and/or the northward migration of the Asian continent linked to the convergence of the Indian and Eurasian plates (and their continental collision).

### A Late Eocene ITCZ signature in monsoonal Asia?

Our results lend support for the existence of a waned continentality in SE Tibet at ~35.5 Ma. However, the evidence for an enhanced hydrological cycle at tropical latitudes during the LED is not explained by a monsoonal intensification since a decline of monsoonal activity during the Late Eocene was inferred from climate simulations^9, 42^. Yet, an underexplored avenue in deep paleoclimate time periods is the ITCZ’s role, through its latitudinal migrations, on rainfall patterns and seasonality at low latitudes^43^. Displacements of the ITCZ have been suggested as the main way in the tropics to respond during global climate change on a variety of time scales. In that sense, any changes in the latitudinal migration of the Late Eocene ITCZ induced by changes in the latitudinal temperature gradient will impact the seasonal wet/dry precipitation ratio experienced in SE Tibet. This hypothesis is fully consistent with the recently published Late Eocene SST data^6^, which showed a paired warming between equatorial and sub-Arctic records coevally with a pronounced austral subpolar cooling 4 Ma prior to the EOT, leading to the inception of a strong meridional temperature gradient between the equator and the Southern Ocean. Hence such a long-term low-latitude warming during the LED would have favored heat accumulation at equatorial and tropical latitudes, which in turn would have fueled reinvigorated ITCZ-induced tropical rainfall in monsoonal Asia. Consistently, Eocene fossil floras of southwest China and northern India imply large latitudinal migrations of a (stronger) Eocene ITCZ inducing a most likely Indonesia-Australia monsoon type in SE Asia, without topographic forcing^44^. Modeling studies also suggested that ITCZ migration extended poleward in the Eocene^42^. In a similar Early Miocene’s synopsis, some authors proposed that the ITCZ paleolatitude was possibly more northerly posited (over SE Asia) at the Oligocene–Miocene boundary^45^. According to these authors, the Mi-1 glaciation at the Oligocene–Miocene boundary would have cooled the Southern Hemisphere relative to the « ice-free » Northern Hemisphere, leading to a shift of more northern (equatorial) peak sea-surface temperatures and a northward drift of the ITCZ. The scenario proposed for the Oligocene-Miocene boundary^45^ may be tentatively explored for the LED in SE Tibet. Cooling of the austral subpolar regions and/or ice sheet formation in Antarctica – which may have occurred as early as around 36.5 Ma in the Weddell Sea^46^ – would cool the Southern Hemisphere, mimicking the seasonal pattern during the austral winter and forcing the ITCZ to shift northwards over monsoonal Asia and SE Tibet. Although the conjecture of an Eocene ITCZ anchored to the Tibetan Plateau was recently debated^47^, a more northern ITCZ over monsoonal Asia, associated with progressive tropical warming and enhanced heat accumulation in the low-latitudes^6^, would arguably favor wetter climatic conditions in our study area (~20°N; Fig. 1); this configuration would create more humidity and less continentality in SE Tibet, as inferred from our sedimentological data documenting persistently wetter conditions around ~35.5 Ma. This Late Eocene climatic pattern over SE Asia does not question the numerous paleoclimatic records documenting a coeval aridification throughout Central Asia^7^. Rather, it does suggest that monsoonal and SE Asia experienced a significant wetter climate during the LED while marked aridity prevailed in Central Asia, due to the persistence of subtropical high pressures throughout most of the year and westerly-dominated surface circulation along the northern margin of the Tibetan Plateau^47^. In addition, our findings suggest that the stepwise retreat of the Tarim Sea – leading to the aridification of Central Asia by reducing moisture supply to the Asian continental interior^48, 49^ – most likely had little impact (if any) on the synoptic-level atmospheric circulation and regional climatic patterns in SE Tibet. Instead, they show that the Asian continental interior and tropical SE Asia experienced different climatic pathways during the doubthouse interval preceding the EOT, along with an increase in the latitudinal precipitation gradient between these two regions. Interestingly, oceanic climatic records show the occurrence of a Late Eocene warming interval bracketed between ~37 and ~35 Ma^1–3^, which should favour global changes in the climate system and an increase of the latitudinal precipitation gradient in Asia, as shown both for Miocene and present-day conditions in Europe^50^. In this regard, our Late Eocene terrestrial sedimentary record in SE Tibet testifies the strong sensitivity of tropical latitudes to oceanic and global climatic changes during the doubthouse interval preceding the EOT.

## Material and Methods

Stratigraphic and sedimentological analyses were conducted near the city of Jianchuan, southwestern China (see Fig. 1 and Supplementary Information). Five stratigraphic sections were performed; the most complete section for the Jiuziyan Fm. is shown on Fig. 3. Stratigraphic logging and correlation was performed using sequential evolution critera (i.e., lithologic and facies evolution, and changes of such evolution vertically and laterally) such as the occurrence of thick-bedded fluvio-palustrine-lacustrine deposits in the Jiuziyan Fm., laterally found over hundreds of meters overlying red alluvial beds. More than one hundred sedimentary samples were collected from these exposures and housed at the Université Claude-Bernard-Lyon1 (Lyon, France). Thin sections were prepared from rock samples at the University Jean Monnet (Saint-Etienne, France) and housed at the Université Claude-Bernard-Lyon1 (Lyon, France). A total of 99 thin sections were prepared for microfacies analyses (for fluvio-palutrine, palustrine and palustrine-lacustrine facies types) under parallel (natural) and polarized light using a Leica microscope LM750P coupled with a Leica digital camera. Thin sections photographs were performed using the software Infinity and further processed in Adobe Photoshop CS6.

### Data and materials availability

All data needed to evaluate the conclusions in the paper are present in the paper and/or in the Supplementary Information. Thin sections were housed at the Université Claude Bernard-Lyon1, Lyon, France.

## Electronic supplementary material

Supplementary Information
