# Supplementary material for: Wet tropical climate in SE Tibet during the Late Eocene
Source: Sci Rep. 2017 Aug 10;7:7809. doi: 10.1038/s41598-017-07766-9 (PMC5552753; doi:10.1038/s41598-017-07766-9)

**Wet tropical climate in SE Tibet during the Late Eocene**

Philippe SORREL^1^*, Inès EYMARD^2^, Philippe-Hervé LELOUP^1^, Gweltaz MAHEO^1^, Nicolas OLIVIER^3^, Mary STERB^1^, Loraine GOURBET^1^, GuoCan WANG^4,5^, Wu JING^4^, Haijian LU^6^, Haibing LI^6^, Xu YADONG^4^, KeXin ZHANG, Kai CAO^4,5^, Marie-Luce CHEVALIER^6^, Anne REPLUMAZ^7^

**Supplementary Information**

**Supplementary Figures**

**Supplementary Figure S1 :**

Geologic cross-sections corresponding to the black amorces on Fig. 2. Geologic formations are shaded when inferred above topography.


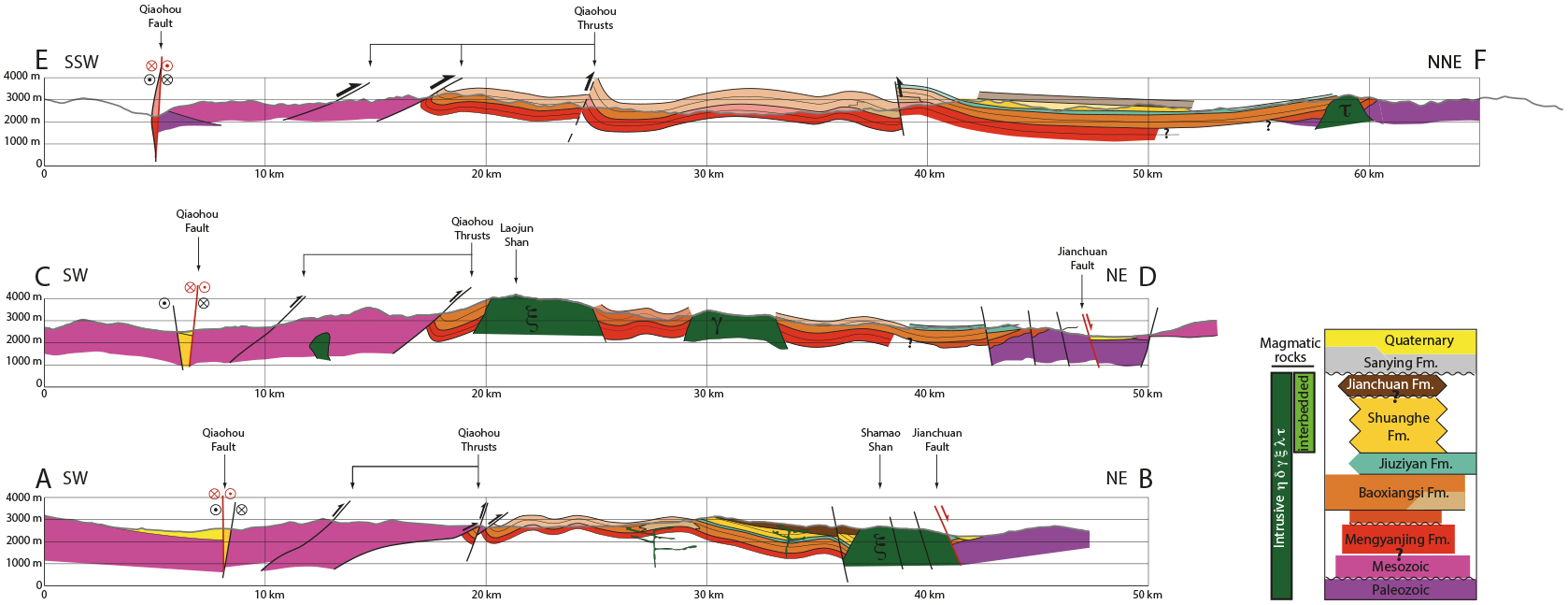


**Supplementary Figure S2**

**Sedimentary facies of the Jiuziyan and Shuanghe Fms**. **See Fig. 2 for location of sites.** (**a**) Debris-flow deposits (matrix-supported conglomerates, facies Cong) consisting of pebbles/cobbles scattered in a clayey matrix. Clasts are subrounded and range from 1–20 cm in size. Note the rare imbrications (arrow). This facies is generated by intensive rainfall in the catchment, resulting in episodic flash floods with high sediment loads (Jiuziyan Fm., site S596). (**b**) Phytoclastic limestone (facies Lphy) (Jiuziyan Fm., site S596). Floatstone of phytoclasts in a micritic matrix, representing high-energy floods in palustrine areas. Shallow areas with hygrophytic plants are sites for boundstone formation (e.g., phytoherm tufa of stems), from where phytoclasts can be reworked during high-energy floods. (**c**) Coal deposits (arrows) regularly interbedded within sandstones (facies Sb), typical of swamp-like deposits (Shuanghe Fm., site S802). Pen for scale. (**d**) Coal layers (facies Co) exploited in local mines (Shuanghe Fm., site S261). (**e**) Trough cross-bedded sandstones (facies Sa) intertwined within sublacustrine marlstones (facies Mar) (Shuanghe Fm., site S263). (**f**) Palustrine-lacustrine facies (facies Lorg). Floatstone of phytoclasts in a micritic matrix. Pen for scale (Shuanghe Fm., site 801).


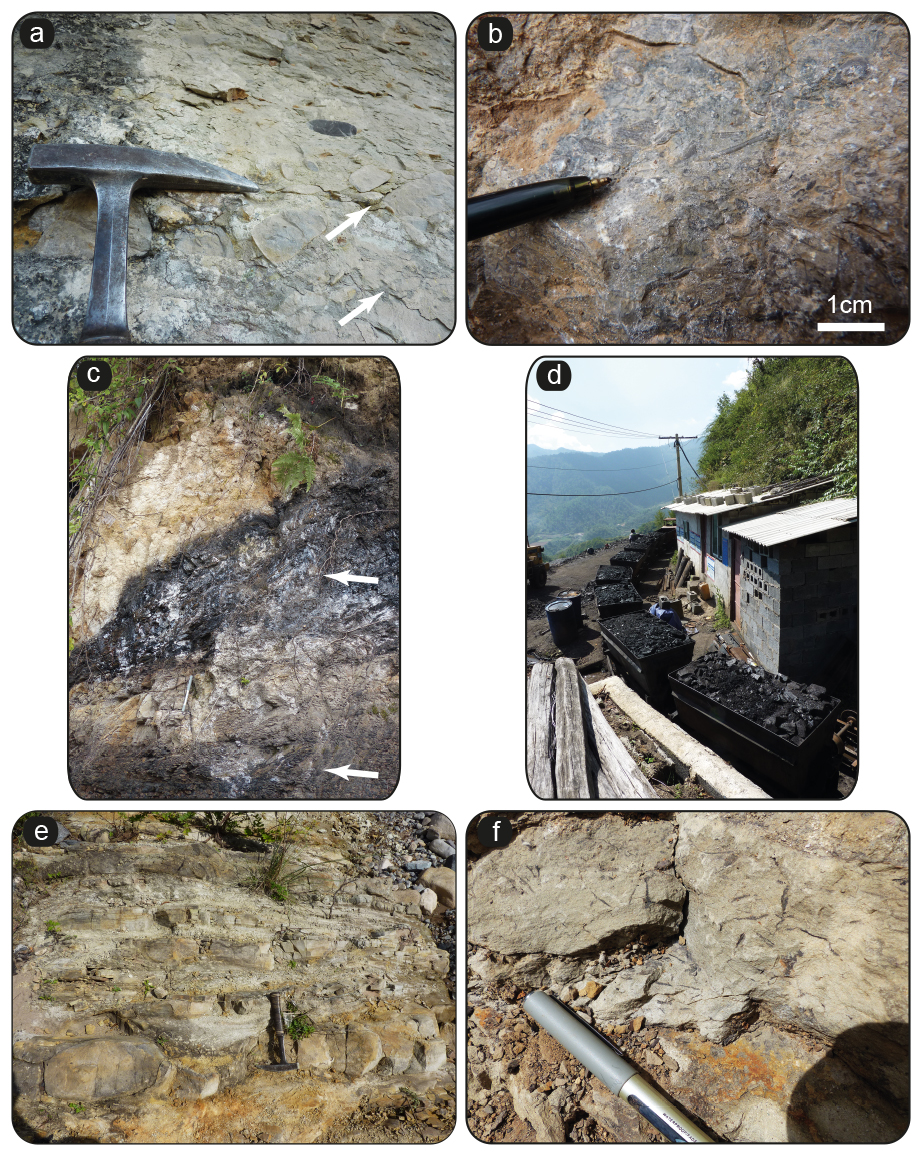


**Supplementary Figure S3**

**Sedimentary facies of the Jiuziyan and Shuanghe Fms**. **See Fig. 2 for location of sites.** (**a**) *In situ* decimeter-sized columnar stromatolites (facies Str). Stromatolites grew in calm, ponded areas of the palustrine-lacustrine system (Jiuziyan Fm., site S254). (**b**) Nodular intraclastic limestone, with well-developed pedogenic features (Jiuziyan Fm., site S254). (**c**) Phytoherm tufa of stems (facies Phs), most likely reeds or rushes (Jiuziyan Fm., site S254). (**d**) Debris-flow deposits consisting of gravels and angular to subangular pebbles in argillaceous calcisiltites (facies Csl) (Jiuziyan Fm., site S254). (**e**) Sandstones (facies Sa) showing climbing-ripple cross laminations, interbedded within grey sublacustrine marls (Jiuziyan Fm., site S596). (**f**) Calcisiltites/calcilutites (facies Csl) (Shuanghe Fm., site 254). (**g**) Fluvial channel-fill deposits showing a fining-up trend (Jiuziyan Fm., site S597). (**h**) Alternation between calcisiltites/calcilutites (facies Csl) and grey lacustrine marls (facies Mar) (Shuanghe Fm., site 254).


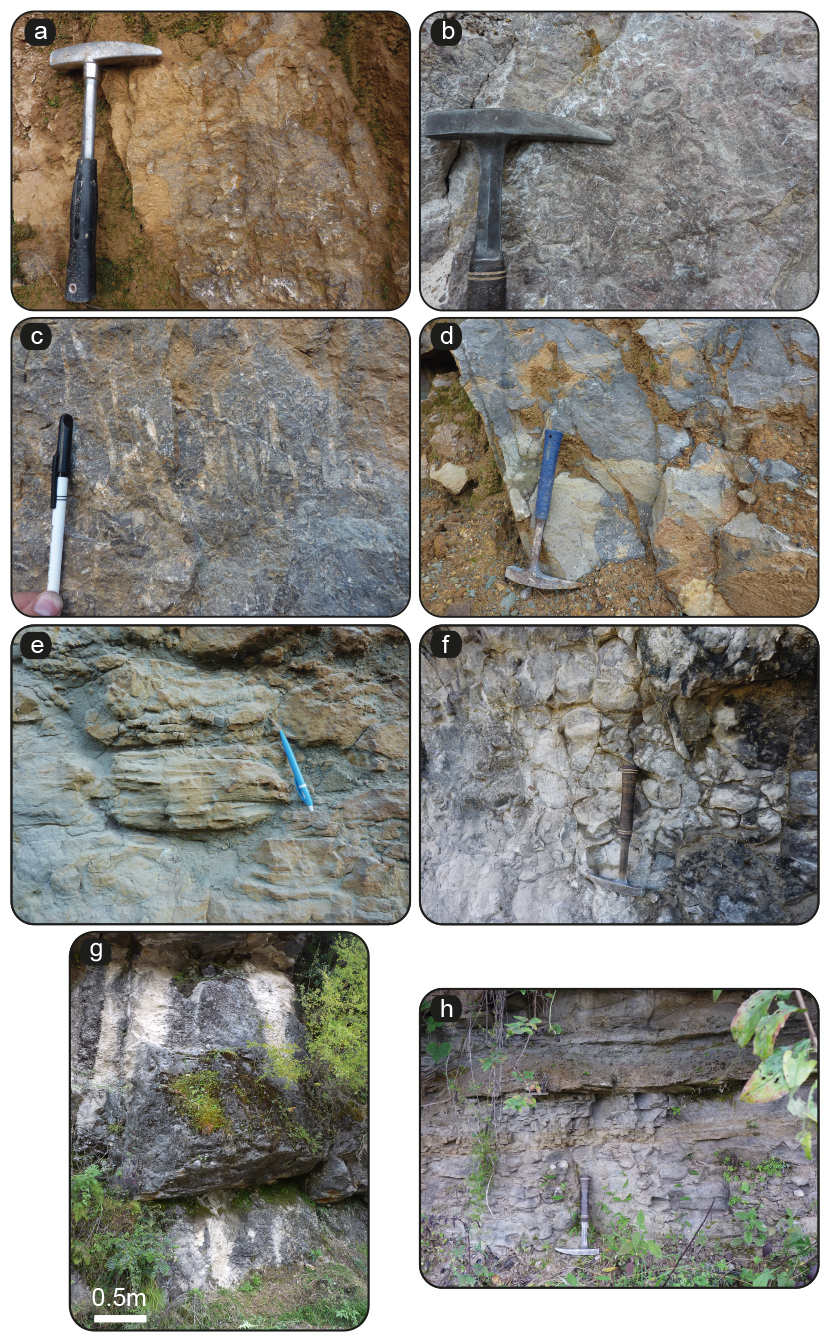


**Supplementary Figure S4**

**Palustrine-lacustrine microfacies of the Jiuziyan Fm. (a–g) and Shuanghe Fm. (h).** **See Fig. 2 for location of sites.** (**a**) Palustrine facies (Facies Phs). Encrusted reed stem showing asymetrical micropeloidal and spar calcite coating. Peloidal and intraclastic limestone with a micritic matrix. The porosity is cemented by coarse spar (Sample J201, site S597). (**b**) Palustrine facies (facies Lgr). Granular limestone composed of intraclasts showing thin micritic coatings (e.g. cortoids), oncoids with poorly concentric coatings and extraclasts containing miliolid forams (arrow). Deposition during high-energy floods in palustrine-lacustrine areas. The grains are randomly distributed within interstitial microspar cement (Sample J158g, site S254). (**c**) Palustrine facies (facies Phs). Micropeloidal and intraclastic limestone. Note the geopetal-infill (way-up structure) within the encrusted reed stem. The porosity is cemented by coarse spar (Sample J158c2, site S254). (**d**) Oncoids (facies Lphy). The nuclei of oncoids can be any carbonate grain available, commonly intraclasts here. Note the thickness of the coatings (>1 mm) (Sample J194, site S596), which consist of an alternation of light and dark laminae. (**e**) Wackestone/packestone containing large extraclasts (i.e., Paleozoic/Mesozoic foraminiferal wackestones/packestones) representative of reworked sources from the catchment during high-energy floods (Sample J171, site S254). (**f**) Caliche (facies Cal). Alveolar septal structures (e.g., microbially-induced calcified root cells) displaying well-developed ovoidal pores and thin micritic laminations (Sample J158d, site S254). (**g**) Oncoid (facies Lim). The nucleus consists of a palustrine intraclast made of alveolar septal structures (Sample J194, site S596). (**h**). Microscopic features of stromatolites, commonly showing a vertical arrangement made of flat, continuous, and/or smooth laminations. Lamination consists of an alternation of light-colored microsparite or spar laminae and dark calcite laminae (facies Str) (Sample J194, site S596).


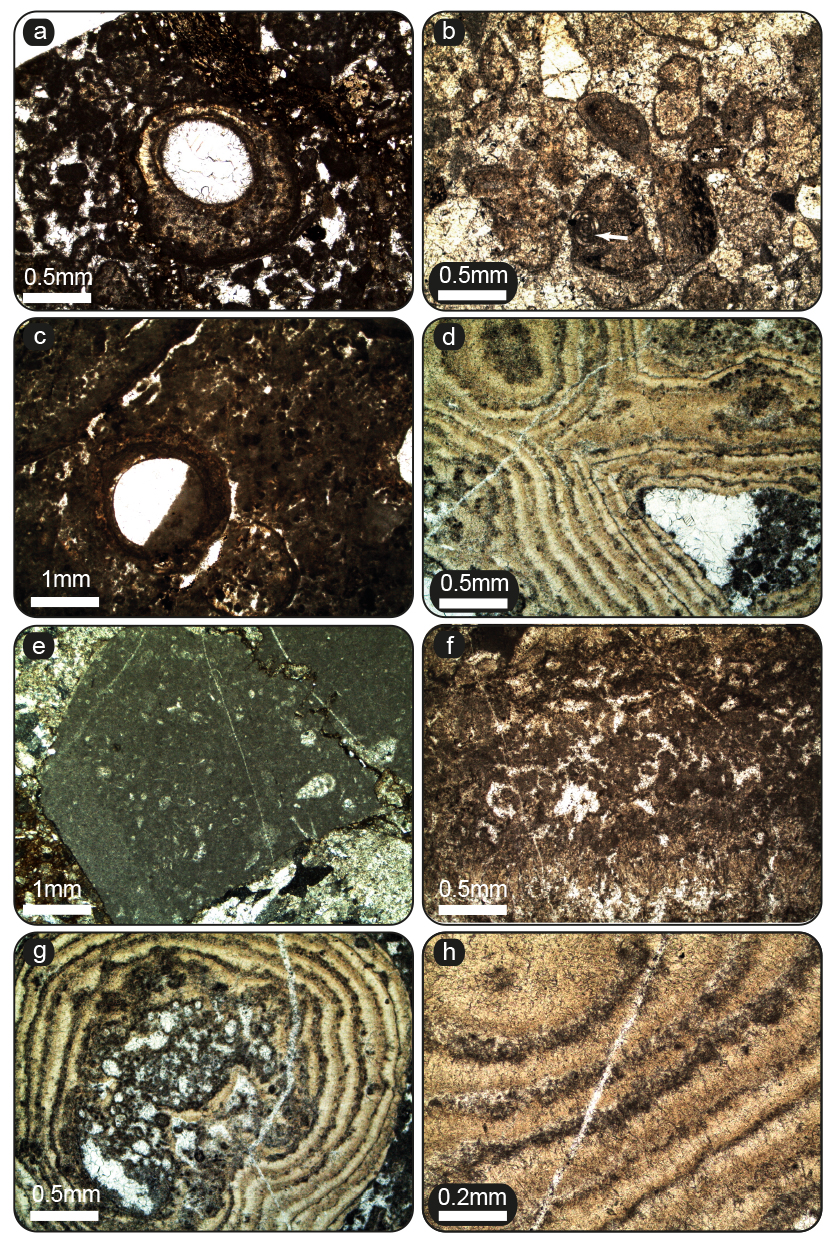


**Supplementary Figure S5**

**Palustrine-lacustrine microfacies of the Jiuziyan Fm. (a–g) and Shuanghe Fm. (h).** **See Fig. 2 for location of sites.** (**a**) Palustrine facies (facies Phs). Note the coating around the macrophyte stem, which consists of an alternation of light and dark laminae (Sample J194, site S596). (**b**) Caliche (facies Cal). Calcified root structures in cross section made of individual calcified root cells. Typical *Microcodium* structure. Note also the alveolar septal structures (arrow) (Sample J225, site S254). (**c**) Caliche (facies Cal). *Microcodium* grains and bunches (e.g., calcified root structures) in a micritic (micropeloidal) matrix (Sample J225, site S254). (**d**) Recristallized gastropod shell in a micritic matrix (facies Lorg). Note the thin micritic inner layer evolving to microspar and coarse spar cement filling the lodges (Sample J255, site S603). (**e**) Coal layer (facies Co) interbedded within limestones (Sample J169, site S261). (**f**) Palynological sample (facies Lorg) showing abundant ligno-cellulosic fragments (phytoclasts) and palynomorphs. Sample collected from organic-rich limestones intertwined with coal layers (Sample J219, site S261). (**g**) Coal layer containing abundant vegetal remains (Sample J169, site S261). (**h**, cross-nicols) Reworked lacustrine facies. Sandstone enriched in mollusc shell debris embedded in a siliciclastic matrix (with abundant quartz grains) (facies Sb), representative of flood events within the lacustrine system (Sample J285B, site S820).


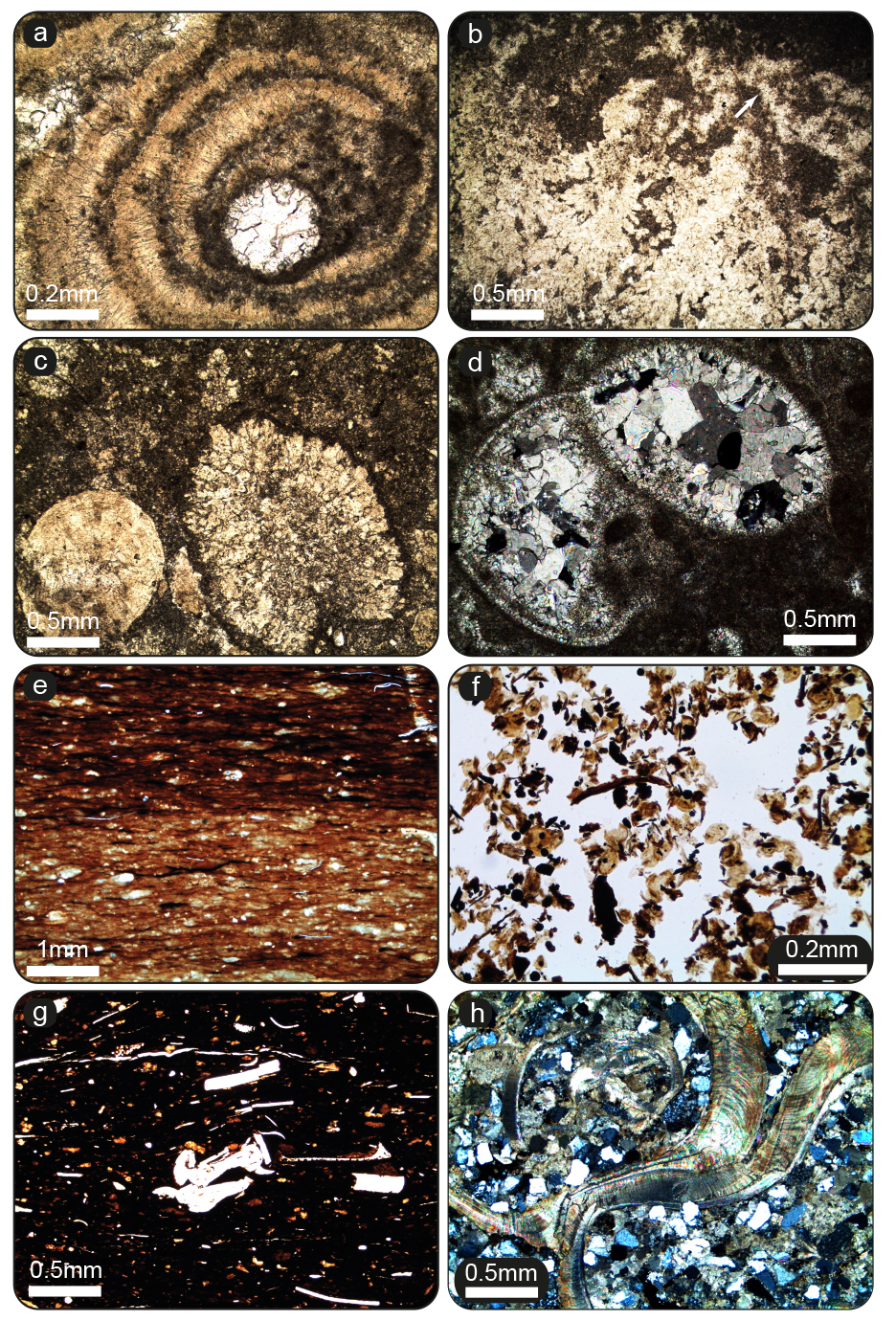


**Supplementary Table 1**

Principal characteristics and interpretation of the facies observed in the Jiuziyan and Shuanghe Fms.

**References**

53. Riding, R. Microbial carbonates : the geological record of calcified bacterial-algal mats and biofilms. *Sedimentology* **47**, 179–214 (2000).

54. Pedley, H.M. Tufas and travertines pf the Mediterranean region : a testing ground for freshwater carbonate concepts and developments. *Sedimentology* **56**, 221–246 (2009).

55. Pedley, H.M., González-Martín, J.A., Ordóñez-Delgado, S. & García Del Cura, M.A. Sedimentology of Quaternary perched springline and paludal tufas : criteria for recognition, with examples from Guadalajara Province, Spain. *Sedimentology* **50**, 23–44 (2003).

56. Freytet, P. & Verecchia E.P. Lacustrine and paslutreine carbonate petrography : an overview. *Journal of Paleolimnology* **27**, 221–237 (2002).


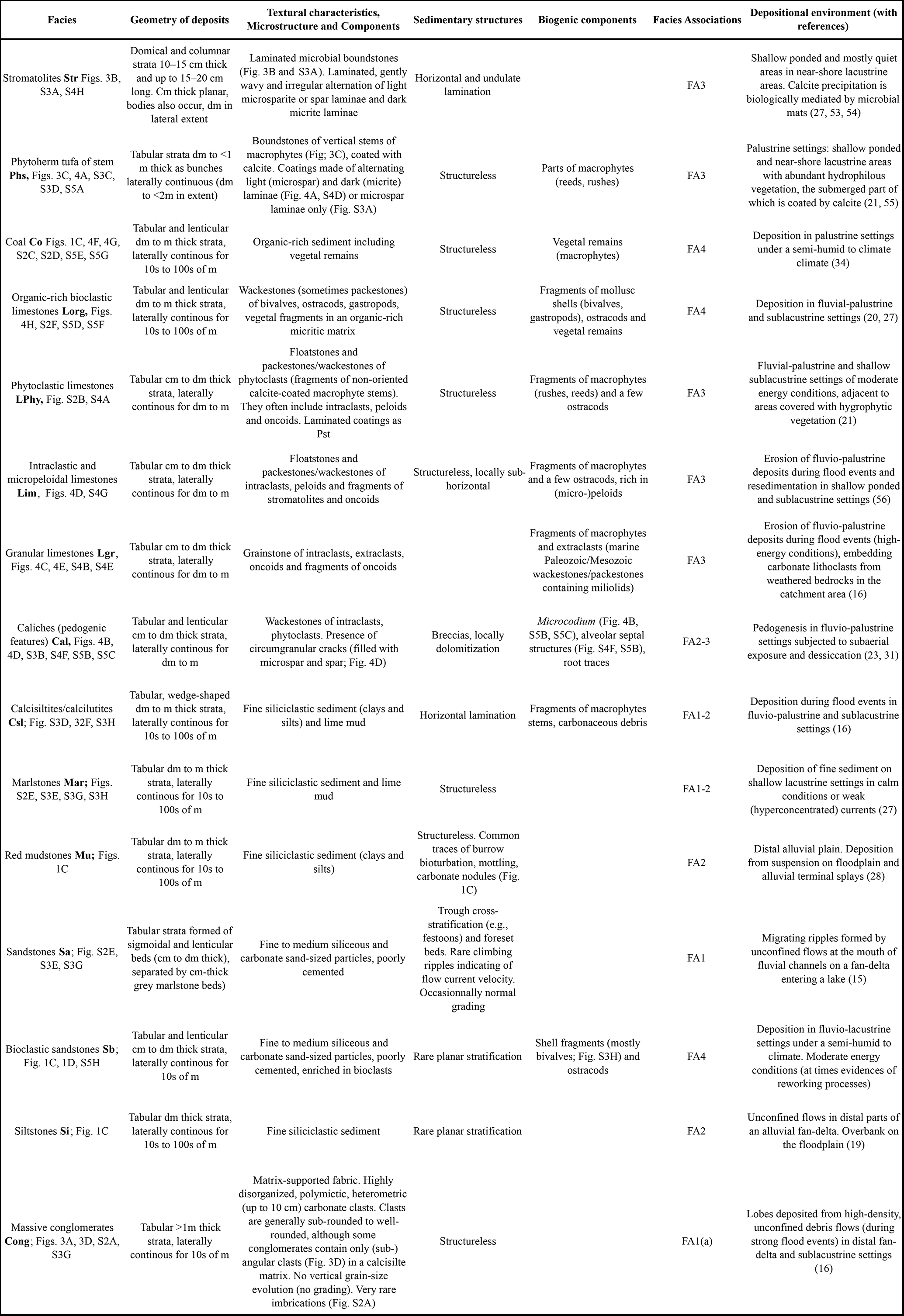

Supplement: Supplementary file 1 — Supplementary Information [file 41598_2017_7766_MOESM1_ESM.docx]
